# Supplementary material for: TFAM Loss Induces Oxidative Stress and Divergent Phenotypes in Glioblastoma Metabolic Subtypes
Source: Int J Mol Sci. 2025 Oct 27;26(21):10446. doi: 10.3390/ijms262110446 (PMC12610520; doi:10.3390/ijms262110446)
Supplement: Supplementary file 1 [file ijms-26-10446-s001.zip › ijms-3895539-supplementary.pdf]

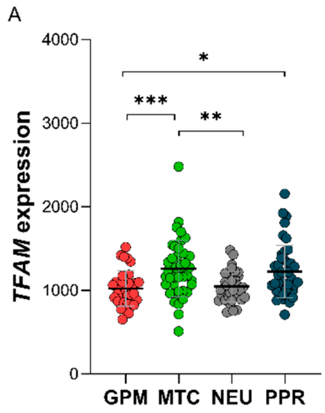

Figure S1. Analysis of TFAM expression in GBM Pathway-based subtypes. (**A**) *In silico* analysis of TCGA astrocytoma database confirming the significant decrease of *TFAM* expression in GBM subtypes according to metabolic axis based on Garofano pathway-based classification demonstrated lower *TFAM* expression in GPM in comparison to MTC, NEU and PPR subtypes. Dots represent each case analyzed, and the line represents the mean ± SD. SD, standard deviation. Statistically significant differences are represented by asterisks: * $p < 0.05$, ** $p < 0.01$, *** $p < 0.001$, **** $p < 0.0001$.
